# Allosteric Regulation at the Crossroads of New Technologies: Multiscale Modeling, Networks, and Machine Learning

**DOI:** 10.3389/fmolb.2020.00136

**Published:** 2020-07-09

**Authors:** Gennady M. Verkhivker, Steve Agajanian, Guang Hu, Peng Tao

**Affiliations:** ^1^Graduate Program in Computational and Data Sciences, Schmid College of Science and Technology, Chapman University, Orange, CA, United States; ^2^Department of Biomedical and Pharmaceutical Sciences, Chapman University School of Pharmacy, Irvine, CA, United States; ^3^Center for Systems Biology, Department of Bioinformatics, School of Biology and Basic Medical Sciences, Soochow University, Suzhou, China; ^4^Department of Chemistry, Center for Drug Discovery, Design, and Delivery (CD4), Center for Scientific Computation, Southern Methodist University, Dallas, TX, United States

**Keywords:** allosteric regulation, multiscale modeling, Markov state models, network analysis, deep learning, reinforcement learning, drug discovery

## Abstract

Allosteric regulation is a common mechanism employed by complex biomolecular systems for regulation of activity and adaptability in the cellular environment, serving as an effective molecular tool for cellular communication. As an intrinsic but elusive property, allostery is a ubiquitous phenomenon where binding or disturbing of a distal site in a protein can functionally control its activity and is considered as the “second secret of life.” The fundamental biological importance and complexity of these processes require a multi-faceted platform of synergistically integrated approaches for prediction and characterization of allosteric functional states, atomistic reconstruction of allosteric regulatory mechanisms and discovery of allosteric modulators. The unifying theme and overarching goal of allosteric regulation studies in recent years have been integration between emerging experiment and computational approaches and technologies to advance quantitative characterization of allosteric mechanisms in proteins. Despite significant advances, the quantitative characterization and reliable prediction of functional allosteric states, interactions, and mechanisms continue to present highly challenging problems in the field. In this review, we discuss simulation-based multiscale approaches, experiment-informed Markovian models, and network modeling of allostery and information-theoretical approaches that can describe the thermodynamics and hierarchy allosteric states and the molecular basis of allosteric mechanisms. The wealth of structural and functional information along with diversity and complexity of allosteric mechanisms in therapeutically important protein families have provided a well-suited platform for development of data-driven research strategies. Data-centric integration of chemistry, biology and computer science using artificial intelligence technologies has gained a significant momentum and at the forefront of many cross-disciplinary efforts. We discuss new developments in the machine learning field and the emergence of deep learning and deep reinforcement learning applications in modeling of molecular mechanisms and allosteric proteins. The experiment-guided integrated approaches empowered by recent advances in multiscale modeling, network science, and machine learning can lead to more reliable prediction of allosteric regulatory mechanisms and discovery of allosteric modulators for therapeutically important protein targets.

## Introduction

Allosteric regulation is an efficient and robust mechanism for molecular communication and signaling in the cell employed by proteins for regulation of activity and adaptability during processes of signal transduction, catalysis, and gene regulation (Monod et al., 1965; Koshland, 1998; Changeux and Edelstein, 2005; Popovych et al., 2006; Changeux, 2012). The recent breakthroughs in nuclear magnetic resonance (NMR) technologies have enabled dynamic studies of large biomolecules at atomic resolution, and are now frequently employed as powerful diagnostic tools of allosteric communications in proteins (Boehr et al., 2006; Jarymowycz and Stone, 2006; Mittermaier and Kay, 2006, 2009; Sprangers et al., 2007; Korzhnev and Kay, 2008; Kalodimos, 2011; Kay, 2011, 2016; Rosenzweig and Kay, 2014; Lisi and Loria, 2016, 2017; Huang and Kalodimos, 2017; Jiang and Kalodimos, 2017). Allosteric molecular events can involve complex cascades of thermodynamic and rapid dynamic changes that occur on different spatial and temporal scales. The thermodynamic-centric energy landscape concepts and conformational selection models of allosteric regulation have gained a considerable prominence in recent years, rooted in the assumption that statistical ensembles of preexisting conformational states and communication pathways are intrinsic to a given protein system (Astl et al., 2019) and allow for modulation and redistribution induced by external perturbations, ligand binding, and mutations (Gunasekaran et al., 2004; Tsai et al., 2008, 2009; del Sol et al., 2009; Csermely et al., 2010, Zhuravlev and Papoian, 2010; Ma et al., 2011; Wrabl et al., 2011; Hilser et al., 2012; Nussinov, 2012; Motlagh et al., 2014; Tsai and Nussinov, 2014; Nussinov and Tsai, 2015; Guo and Zhou, 2016; Liu and Nussinov, 2016; Astl et al., 2019). Conformational dynamics redistributions in the absence of appreciable structural transformations are the hallmark of the “entropy-driven” allosteric mechanisms in which allosteric interactions can be mediated through alterations of functional motions and rebalancing of rigid and flexible protein regions (Cooper and Dryden, 1984; Stevens et al., 2001; Dam et al., 2002; Kern and Zuiderweg, 2003; Frederick et al., 2007; Tzeng and Kalodimos, 2009, Nesmelova et al., 2010; Kalodimos, 2011, 2012; McLeish et al., 2013; Li et al., 2014; Buchenberg et al., 2017; Stock and Hamm, 2018; Wodak et al., 2019). The quantitative elucidation of these highly dynamic and often elusive processes continues to present formidable technical and conceptual challenges. Despite significant advances, the quantitative characterization and prediction of functional allosteric states, interactions and mechanisms continue to present highly challenging problems in the field. The fundamental biological importance and complexity of these processes require innovative computational and experimental approaches that can advance current understanding of allosteric regulatory processes. A systematic interdisciplinary effort is needed to leverage the burgeoning knowledge about allosterically regulated proteins to develop robust experiment-informed computational tools for atomistic prediction of allosteric mechanisms. In this review we discuss and analyze how recent advances in biophysical simulations and network science can be integrated with NMR spectroscopy experiments and leverage the rising power of machine learning (ML) approaches to enable the reliable quantitative characterization of allosteric regulation mechanisms and facilitate allosteric drug discovery. We discuss in details computational strategies that leverage biophysical and network-based modeling with NMR experiments for characterization and probing of allosteric regulatory mechanisms. The review also critically discusses advantages and limitations of emerging approaches including Markovian modeling and the information-theoretical analysis of dynamic flows in allosteric networks in addressing present challenges and open questions of allosteric regulation mechanisms.

## Network-Based Approaches in Studies of Allosteric Regulation Mechanisms

It has been recognized that allosteric regulation is a global property of protein systems that can be described by the residue interaction networks in which the effector binding initiates a cascade of coupled fluctuations propagating through the network and eliciting long-range functional responses at distal sites (Atilgan et al., 2004; Brinda and Vishveshwara, 2005, 2010; del Sol and O'Meara, 2005; Bode et al., 2007; Sethi et al., 2009; Vijayabaskar and Vishveshwara, 2010; Csermely et al., 2013; Di Paola and Giuliani, 2015; Dokholyan, 2016). The graph-based network approaches have offered a simple and effective formalism for describing allosteric interactions, where the dynamic fluctuations are mapped onto a graph with nodes representing residues and edges representing weights of the measured dynamic properties. The network-centric methods have represented a powerful complementary strategy to physics-based landscape models of protein dynamics by quantifying global functional changes (Vendruscolo et al., 2002; Atilgan et al., 2004; Brinda and Vishveshwara, 2005, 2010; Ghosh and Vishveshwara, 2007, 2008; Hansia et al., 2009; Bhattacharyya and Vishveshwara, 2011; Ghosh et al., 2011; Csermely et al., 2012; Gasper et al., 2012; Bhattacharya and Vaidehi, 2014; General et al., 2014; Dokholyan, 2016; Adhireksan et al., 2017), identifying key functional centers and allosteric communication pathways (Verkhivker et al., 2002; del Sol and O'Meara, 2005; del Sol et al., 2006; Sethi et al., 2009, 2013; Vijayabaskar and Vishveshwara, 2010; Rivalta et al., 2012; Vanwart et al., 2012; Farabella et al., 2014; Di Paola and Giuliani, 2015; Kalescky et al., 2015, 2016; Hertig et al., 2016; Ricci et al., 2016; Stolzenberg et al., 2016; Palermo et al., 2017; Zhou et al., 2017, 2019a,b; Liang et al., 2019; Li et al., 2019). Recent years have witnessed the proliferation of numerous computational tools for predicting allosteric pathways and communications in proteins (Ming and Wall, 2005, 2006; McClendon et al., 2009; Tehver et al., 2009; Mitternacht and Berezovsky, 2011; Bowman and Geissler, 2012; Panjkovich and Daura, 2012, 2014; Goncearenco et al., 2013; Kaya et al., 2013; Stetz and Verkhivker, 2017). The network studies have also suggested that rapid signal transmission of allosteric interactions through small-world networks encoded in protein folds may be a universal signature encoded in protein families (Tsai et al., 2009; Di Paola and Giuliani, 2015). Significant bodies of computational and experimental studies have shown that integration of network-based approaches with structural and biochemical studies can provide a robust platform for further exploration and atomistic characterization of allosteric states and regulatory mechanisms controlled by allostery.

Functional residues in residue networks are often connected via strong evolutionary relationships (Lockless and Ranganathan, 1999; Suel et al., 2003; Halabi et al., 2009; Aguilar et al., 2012; McLaughlin et al., 2012; Simonetti et al., 2013). Coevolution of protein residues can reflect correlated functional dynamics of these sites in mediating residue-residue contacts (Socolich et al., 2005), protein folding transitions (Morcos et al., 2011), and allosteric signaling in protein complexes (Wang et al., 2019). Coevolving residues could also form direct communication paths in the interaction networks with connections weighted according to dynamic couplings and coevolutionary interaction strengths between nodes (Chakrabarti and Panchenko, 2009, 2010; Nishi et al., 2011). Dynamic and coevolutionary residue correlations may also act as synchronizing forces that determine modular organization of allosteric interaction networks and enable efficient allosteric regulation (Stetz and Verkhivker, 2017). These results have motivated the development of novel community-based methods for modeling ensembles of allosteric communication pathways in protein structures (Tse and Verkhivker, 2015a,b; Verkhivker et al., 2016; Stetz and Verkhivker, 2017). Using this computational framework, it was found that efficient allosteric communications in various signaling proteins could be controlled by structurally stable functional centers that exploit dynamically coupled residues in their local communities to propagate cooperative structural changes. The important revelation of these studies was that dynamic and evolutionary residue correlations may act as synchronizing forces to enable efficient and robust allosteric regulation.

Examining proteins as dynamic regulatory machineries that fluctuate between functional allosteric states and modulated by ligand binding or mutations is critical to understanding the molecular principles of signaling in the cell. Computational studies of allosteric regulation in signaling proteins have led to important mechanistic insights, better atomistic understanding of complex regulatory processes and continuous integration with structural and functional experiments. A variety of computational approaches have been extensively explored in investigations of allosteric mechanisms in protein kinases. These studies included experiment-guided structural modeling and protein folding analysis (Levinson et al., 2006; Zhang et al., 2006; Kornev et al., 2008; Han et al., 2011; Jura et al., 2011; Shan et al., 2011, 2012, 2013; Taylor and Kornev, 2011; Tzeng and Kalodimos, 2011; Levinson and Boxer, 2012, 2014; Taylor et al., 2012a,b; Meharena et al., 2013; Shaw et al., 2014; Shukla et al., 2014; Kornev and Taylor, 2015; Schulze et al., 2016; Narayanan et al., 2017; Levinson, 2018; Ruff et al., 2018), molecular simulations and free energy computations (Yang and Roux, 2008; Dixit and Verkhivker, 2009, 2011a,b; Yang et al., 2009; Arkhipov et al., 2013; Lin and Roux, 2013; Lin et al., 2013, 2014; Dixit and Verkhivker, 2014; Meng and Roux, 2014; Fajer et al., 2017; Kim et al., 2017; Meng et al., 2017), and network modeling (James and Verkhivker, 2014; Tse and Verkhivker, 2015a,b,c; Czemeres et al., 2017; Stetz et al., 2017; Astl and Verkhivker, 2019a,b). By examining residue interaction networks in protein kinases a unifying mechanistic model of allosteric coupling between the ATP-binding and substrate binding sites conserved among kinases was proposed (Tse and Verkhivker, 2015a,b,c; Stetz et al., 2017). A theoretical framework for rationalizing binding preferences of the kinase inhibitors was developed establishing the relationships between ligand binding and modulation of the residue interaction networks (Tse and Verkhivker, 2015a,b,c). Atomistic modeling of the ABL kinase regulation using a combination molecular dynamics (MD) simulations, structural perturbation methods and network-centric analysis (Astl and Verkhivker, 2019a,b) has provided evidence of allosteric interactions and communication pathways in the ABL interaction networks that supported and explained the underlying mechanisms proposed in the pioneering NMR studies (Saleh et al., 2017).

Computational studies of allosteric regulation in molecular chaperones Hsp90 and Hsp70 have also been instrumental to the progress in the field by complementing biochemical experiments and providing a detailed dynamic view of the functional cycle and mechanisms (Colombo et al., 2008; Morra et al., 2009, Verkhivker et al., 2009; Morra et al., 2010, 2012; Matts et al., 2011a,b; Chiappori et al., 2012, 2016; Dixit and Verkhivker, 2012; Lawless et al., 2013; Verkhivker, 2014, 2018a,b; Paladino et al., 2015; Stetz and Verkhivker, 2015, 2016, 2017, 2018; Czemeres et al., 2017; Stetz et al., 2017). Using a network-based formalism of allostery, computational studies have captured NMR-observed direction-specific nature of signal propagation pathways in the Hsp70 chaperone (Stetz and Verkhivker, 2015, 2017).

Studies of allosteric mechanisms have indicated that integration of experiment-informed molecular simulations with network-based formalisms of allostery may provide a convenient and powerful platform for atomistic characterization of allosteric states and regulatory mechanisms. The lessons from studies of signaling proteins including protein kinases and molecular chaperones have suggested that allosteric regulation mechanisms can proceed via a non-trivial and often elusive combination of the three classical models of allostery: induced fit, conformational selection, and dynamic allostery. Computational modeling and atomistic simulations of protein systems and functional assemblies have shown that allosteric mechanisms may not necessarily imply a simple switching between the crystal structures of the inactive and active states, but often represent a complex regulatory machinery in which binding and external perturbations could give rise to a spectrum of functionally relevant and yet often hidden allosteric conformations exhibiting a range of activity levels.

## Allosteric Regulation and Detecting Allosteric States Through Integration of NMR Experiments and Computational Modeling

The growing number of high-resolution crystal structures and wealth of structural information about protein systems have had an enormous impact on computational and simulation approaches, facilitating development of knowledge-based methods and advanced sampling techniques. However, allosteric functional states in proteins are often highly dynamic and short-lived representing low populated, high energy states that are rarely directly observed in X-ray crystallography experiment. A large amount of conformational sampling is typically needed to uncover and isolate high-energy functional states simulations. For instance, cryptic (or hidden) allosteric sites sporadically appear during conformational transitions of a protein in the presence of a bound ligand. These hidden allosteric sites are invisible in crystal structures and can be detected due to the stabilization of the low-populated, higher-energy conformation by certain compounds. Even with the advanced sampling techniques and enormous computer power that is now available, the experimental validation and confirmation of allosteric states represents the key component to ensure robust quantitative modeling and analysis of allosteric mechanisms.

NMR spectroscopy is a powerful method for studying protein dynamics and allosteric mechanisms by probing multiple time scales and detecting residue-specific conformational changes associated with ligand binding (Boehr et al., 2006; Jarymowycz and Stone, 2006; Mittermaier and Kay, 2006, 2009; Sprangers et al., 2007; Korzhnev and Kay, 2008; Kalodimos, 2011; Kay, 2011, 2016; Rosenzweig and Kay, 2014; Lisi and Loria, 2016, 2017; Huang and Kalodimos, 2017; Jiang and Kalodimos, 2017). The micro- to milli-second time scale protein motions measured in relaxation-dispersion experiments can provide information about the distribution of conformational states and thermodynamics and kinetics of allosteric protein changes. Protein dynamics can also be investigated by NMR methods other than traditional relaxation experiments. Residual dipolar couplings are sensitive to motions occurring across a vast time scale, ranging from seconds to faster than nanoseconds. Conformational changes in isotopically labeled proteins upon ligand binding can be detected by two-dimensional heteronuclear single quantum coherence (HSQC) spectroscopy for large protein systems (Sprangers et al., 2007; Korzhnev and Kay, 2008). Chemical shift mapping of protein residues upon ligand binding provides a specific and precise fingerprint of allosteric propagation effects that allows to detect site-specific binding responses, characterize pathways of allosteric communication and differentiate between competitive and allosteric inhibitor binding (Grutsch et al., 2016; Berjanskii and Wishart, 2017; Krivdin, 2017; Nerli et al., 2018). The NMR technologies have enabled structural studies of conformational dynamic processes at atomic resolution and are used to identify coupled networks and communication pathways in allosteric proteins (Swain and Gierasch, 2006; Smock and Gierasch, 2009; Shi and Kay, 2014; Grutsch et al., 2016). Relaxation dispersion NMR methods have enabled detection and characterization of rare and energetically excited conformational states that play significant role in dynamic activation of protein function and allosteric mechanisms (Vallurupalli et al., 2012; Kalbitzer et al., 2013; Munte et al., 2013; Sekhar and Kay, 2013, 2019; Williamson and Kitahara, 2019). Characterization of low-lying excited states of proteins by high-pressure NMR under equilibrium conditions can allow for detection of reversible transitions that are functionally relevant, providing means for probing dynamic energy landscapes of allosteric mechanisms (Kalbitzer et al., 2013; Williamson and Kitahara, 2019). High-pressure NMR can help to identify these conformations, including low populated functional states, and characterize their energies and kinetics of conformational changes (Williamson and Kitahara, 2019). By measuring redistributions in the conformational entropy, pressure-dependent chemical shifts can help to sequester low-populated functional states (Kalbitzer et al., 2013; Munte et al., 2013; Williamson and Kitahara, 2019).

**Figure 1:**
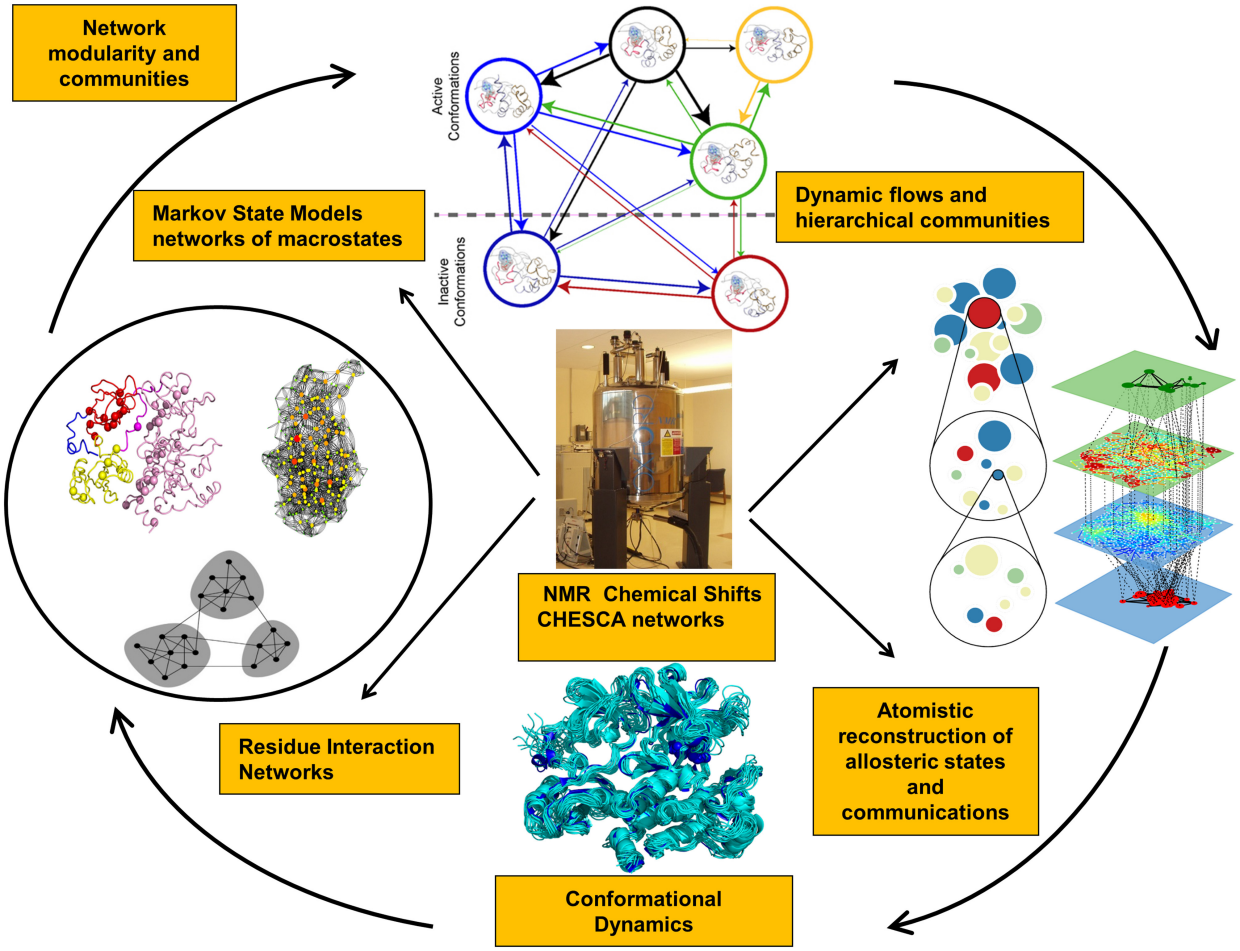
Integration of NMR experiments and computational approaches for experiment-guided analysis of allosteric states and mechanisms.

Recent years have witnessed the development of various approaches that investigate NMR chemical shift perturbations to identify potential allosteric networks and structural dynamics in proteins (Selvaratnam et al., 2011, 2012; Robustelli et al., 2012; Cembran et al., 2014). NMR chemical exchange saturation transfer (CEST) experiments can provide adequate characterization of slower exchange processes, identify invisible states, and slow conformational exchange (Long et al., 2014; Anthis and Clore, 2015; Yuwen et al., 2017). NMR chemical shift covariance (CHESCA) and projection (CHESPA) analyses can identify blocks of dynamically coupled residues collectively forming allosteric interaction networks (Selvaratnam et al., 2011, 2012; Boulton et al., 2014, 2018; Boulton and Melacini, 2016). Allosteric proteins subjected to specific perturbations (ligand binding, mutations) cause residues that belong to the same effector-dependent allosteric network to exhibit a concerted response signal. CHESCA approach can detect patterns of correlated changes in the chemical shifts between apo and holo states due to perturbations and isolate allosterically coupled regions (Figure 1). This method is particularly effective in detecting allosteric networks within dynamic and partially unstructured regions (Boulton and Melacini, 2016; Boulton et al., 2018). NMR chemical shift perturbations have been recently used in combination with Markov model network analysis to reveal the dynamic flow of communication between allosteric communities in the protein kinases (Aoto et al., 2016). NMR-guided computational modeling can leverage CHESCA approach for computation of the chemical shift correlation matrices in the known allosteric states obtained using crystal structures of complexes with allosteric ligands. The experimental NMR chemical shifts can guide molecular simulations and network analysis by reporting on blocks of dynamically coupled residues forming allosteric interaction networks. Through integration of these experimental data into accelerated atomistic simulations, a more detailed mapping of the functional landscapes and relevant allosteric states can be achieved.

Protein systems can be efficiently simulated on longer time scales by accelerated meta-dynamics approaches (Limongelli et al., 2013; Palazzesi et al., 2013, 2017; Sutto and Gervasio, 2013; Bonomi and Camilloni, 2017; Kuzmanic et al., 2017; Yang et al., 2018; Brotzakis and Parrinello, 2019) where the experimental and computed NMR chemical shifts (Shen and Bax, 2010; Han et al., 2011) are often used to determine collective variables (Granata et al., 2013; Xia et al., 2013; Palazzesi et al., 2017). NMR chemical shifts can be also evaluated using structure-based CamShift approach (Kohlhoff et al., 2009) with collective variables defined as the difference between experimental and calculated chemical shifts. These NMR-guided simulation techniques have enabled adequate sampling of the conformational space and robust structure reconstruction using experimental constraints (Robustelli et al., 2010; Cavalli et al., 2011; Granata et al., 2013). NMR chemical shift observables can be also used in combination with other collective variables in meta-dynamics simulations to guide the efficient exploration of allosteric states and functional transitions (Kimanius et al., 2015; Ansari et al., 2016).

A combination of powerful and expensive NMR spectroscopy equipment, biophysical techniques and protein expression platforms is often required to obtain structures of allosteric states for protein systems and experimental validation of short-lived hidden functional conformations. Despite unique abilities of NMR spectroscopy to detect highly dynamic events and examine conformational landscapes of allosteric proteins, the NMR applications for high-resolution reconstruction of allosteric states are still fairly limited owing to complexity and cost of these experiments. Hence, development of novel research strategies based on innovative integration of NMR spectroscopy and experiment-guided simulation approaches become especially important and clearly represent the most promising avenue for further explorations going forward.

## Markov State Models in Studies of Allosteric Regulation

Given the complexity of thermodynamic and kinetic factors underlying allosteric regulatory events, the information-based theory of signal propagation (Chennubhotla and Bahar, 2006, 2007; Chennubhotla et al., 2008) and stochastic Markov state models (MSMs) (Prinz et al., 2011; McGibbon et al., 2014; Pande, 2014; Shukla et al., 2015, 2017; Wu et al., 2016; Husic and Pande, 2018) have become increasingly useful states-and-rates network models with the continuously developing open source software infrastructure (Cronkite-Ratcliff and Pande, 2013; Bowman, 2014; Bowman and Noe, 2014; Harrigan et al., 2017). The MSMs have been successfully adopted for describing the transitions between functional states during allosteric events (Bowman et al., 2015; Hart et al., 2016; Sengupta and Strodel, 2018). Combined with MD simulations, MSM approaches can provide connectivity maps of states on the free energy landscape, estimate the effect of allosteric perturbations on the conformational equilibrium, and rigorously describe kinetics of allosteric transitions. Recent advances in the field have highlighted how MSM tools can help to recognize structural and dynamic patterns of conformational ensembles, identify functional allosteric states hidden in the conformational ensembles and reconstruct allosteric mechanisms (Sengupta and Strodel, 2018). Markov models have been employed for understanding of the reaction mechanisms, thermodynamics and free-energy landscape population shifts, the hierarchy of timescales and the structural basis of allosteric events (Prinz et al., 2011; Pande, 2014; Shukla et al., 2015, 2017; Zhou et al., 2017, 2019a,b).

When combined with appropriate general coordinates, MSM could be a very powerful tool to reveal intrinsic states of the proteins (Malmstrom et al., 2015). The important component of the MSM approach in studies of allosteric systems is the employment of robust dimensionality reduction techniques to identify experimentally-informed collective variables that can enhance sampling and provide efficient detection and separation of functional allosteric states. Dimension reduction is often performed using time-lagged independent component analysis (TICA) (Schwantes and Pande, 2014; Perez-Hernandez and Noe, 2016; Noe and Clementi, 2017; Olsson et al., 2017). In these approaches, the simulation samples can be divided into substates assuming that conformations within each substate share kinetic similarity and could interconvert rapidly (Bowman et al., 2009; Zhou and Tao, 2018; Zhou et al., 2018a,b). t-SNE method was recently developed as a dimensionality reduction method with minimum structural information loss revealing that both one-dimensional (1D) and two-dimensional (2D) models of t-SNE method are superior to other tools in distinguishing functional states of allosteric proteins (Zhou et al., 2018a,b). MSMs and transition network models are widely applied to extract kinetic descriptors from equilibrium simulations. Directed Kinetic Transition Network (DKTN) which is a graph representation of a master equation was developed for describing non-equilibrium kinetics in allosteric proteins (Zhou et al., 2019a,b). Markov modeling studies have also examined the timescales and intra-molecular pathways implicated in allostery by introducing master equation-based approach for allostery by population shift (Long and Bruschweiler, 2011). Another study employed a graph-theoretic approach and Markov stability analysis for modeling of signaling pathways and characterization of allosteric sites (Amor et al., 2014).

Current allosteric models have suggested that conformational and dynamical distribution phase space accessible for allosteric interactions in proteins is much larger than the experimentally visible landscapes provided through crystallographic and NMR experiments. As a result, external perturbations, such as mutations and/or ligand binding that could significantly affect conformational space and dynamic distribution of allosteric proteins and can be employed as probes to explore functional consequences of allosteric phenomena. The recently developed Rigid Residue Scan (RRS) simulation method has been shown as effective tool to perturb protein dynamics and assess both conformational and dynamical redistributions in allosteric systems (Kalescky et al., 2015, 2016). Using the RRS method, the predictive models for light-oxygen-voltage-sensing (LOV) domains allostery have been developed that identified the experimentally verified mutants with distinctive allosteric regulatory effects. The results of this analysis have suggested how manipulating functional regions with light in LOV proteins could link chemistry and allostery, providing a path for rational engineering of LOV ontogenetic tools.

## Exploiting Allosteric Mechanisms and Cryptic Binding Sites for Discovery of Allosteric Modulators

Multiscale simulations and MSM approaches have shown that allosteric mechanisms may not necessarily imply a simple two-state switch between the major inactive and active states, but often represent a dynamic multilayered regulatory machine in which binding and external perturbations could give rise to a discrete spectrum of functionally relevant and yet often hidden allosteric conformations exhibiting a range of activity levels. Experiment-informed Markovian modeling studies have shown a promise in adequately describing the hierarchy of allosteric states by recognizing structural and dynamic patterns of conformational ensembles and identifying functional allosteric states that are hidden in the crystal structures of allosteric proteins. Discovery of multiple hidden allosteric sites by combining Markov state models and experiments has been advanced and applied for antibiotic target TEM-1 β-lactamase (Bowman et al., 2015). Bowman et al. used MSM approach of a ligand-free protein to identify allosteric sites based on several signatures of collective dynamics, namely the presence of a pocket in a significant fraction of the population and the presence of correlated motions between the newly discovered pocket and the active site which provides means for allosteric communication between distant sites. The central to this pioneering work is a close integration with labeling experiments on TEM-1 β-lactamase that were performed to test the existence of hidden allosteric sites as feasible targets for allosteric drug design (Bowman et al., 2015). These illuminating studies have shown for the first time the power of integrated tools to identify, characterize and exploit hidden allosteric sites, highlighting the robust nature of Markov modeling tools in guiding the experiments. It has been argued that the wealth of thermodynamic, kinetic and structural data derived from MSMs can guide further development of experimental tools for discovery of hidden allosteric states and invisible cryptic allosteric binding sites.

The results suggest there are many undiscovered hidden allosteric sites that can be characterized and targeted with rational drug design (Cimermancic et al., 2016; Oleinikovas et al., 2016; Beglov et al., 2018; Kuzmanic et al., 2020). The hidden allosteric sites are invisible in crystal structures and cryptic sites can emerge as a result of stabilization of rare, high-energy states by small fragment probes. The allosteric mechanisms of cryptic site formation may involve a delicate interplay between induced-fit and conformational selection that can be modeled using elaborate replica-exchange sampling techniques (Oleinikovas et al., 2016). Collectively, experiment-informed multiscale simulation studies have shown that these tools can adequately describe complexity and stochasticity that underlies the thermodynamics and hierarchy of allosteric states and the molecular basis of allosteric mechanisms.

Recent advances in understanding allosteric regulation and activation mechanisms of therapeutic signaling proteins such as protein kinases have fueled unprecedented efforts to discover targeted allosteric inhibitors. Allosteric kinase inhibitors do not compete with ATP and could be more selective by binding to the regulatory sites outside of the ATP binding site (Dar and Shokat, 2011). Allosteric kinase inhibitors can improve target specificity and play an important role in the precision medicine initiative in oncology. NMR and X-ray crystallography studies have revealed a detailed atomistic picture of allosteric regulation in many protein kinases, showing how interacting signaling modules form a multilayered regulatory mechanism that exploits various allosteric switch points powered by binding or phosphorylation at different sites of the regulatory kinase complexes (Saleh et al., 2017). Recently discovered allosteric inhibitors of the ABL kinase GNF-2, GNF-5, and ABL001 (Asciminib) bind to the allosteric pocket near the C terminus of the ABL kinase domain stabilizes the inactive conformation of the kinase (Adrian et al., 2006; Zhang et al., 2010). Using solution NMR, X-ray crystallography, mutagenesis and hydrogen exchange mass spectrometry, it was shown that allosteric inhibitors can induce long-range structural and dynamic changes in the remote ATP-binding site (Adrian et al., 2006; Zhang et al., 2010; Wylie et al., 2017; Schoepfer et al., 2018). While the field of kinase inhibitors has enjoyed unprecedented success manifested in multiple FDA approved drugs, the development of allosteric kinase activators has been lagging behind. The mechanisms underpinning allosteric action of kinase activators can proceed by destabilization of the inactive state, stabilization of the active state, facilitating of the active state, and dynamic responses to phosphorylation in regulatory sites (Dar and Shokat, 2011; Fang et al., 2013; Hu et al., 2013; Cowan-Jacob et al., 2014). Structural and biochemical studies of allosteric inhibitors and activators of ABL kinase have indicated that structural environment near the allosteric pocket can serve as a sensor of ligand binding, triggering either stabilization of the inactive state or large conformational shift and activation. Furthermore, synergistic actions of allosteric and ATP competitive inhibitors have provided evidence that binding can perturb dynamics at distal regions and elicit ligand-specific communication between binding sites. Computational studies have detailed how allosteric inhibitors and activators may exert a differential control on allosteric signaling between binding sites (Astl and Verkhivker, 2019a). It was found that while inhibitor binding can strengthen the inhibitory ABL state and induce allosteric communications directed from the allosteric pocket to the ATP binding site, DPH activator may induce a more dynamic kinase state and preferentially activate allosteric couplings between the ATP and substrate binding sites (Astl and Verkhivker, 2019a).

By combining computational and experimental approaches a significant progress has been made in discovery of allosteric modulators of Hsp90 and Hsp70 chaperones. Recent studies have demonstrated that the C-terminal domain (CTD) of Hsp90 is important for dimerization of the chaperone and contains a second nucleotide binding site (Marcu et al., 2000a,b). The bacterial gyrase inhibitor novobiocin, a member of the coumeromycin family of antibiotics, is an Hsp90 antagonist that was found to inhibit a second ATP binding site at the C-terminus. Novobiocin binds the C-terminal nucleotide pocket and displaces both ATP and geldanamycin, and inhibits Hsp90's function (Marcu et al., 2000a,b). The principal advantage of C-terminal inhibition over N-terminal inhibition is the lack of a heat shock response upon ligand binding at the C-terminus of Hsp90. The first compounds that clearly differentiated between the C-terminus of Hsp90 and DNA gyrase, and converted a well-established gyrase inhibitor into a selective Hsp90 inhibitor were initially reported by Donnelly and Blagg (2008), Matts et al. (2011a), Matts et al. (2011b), Garg et al. (2016, 2017a,b), Hall et al. (2016), Khandelwal et al. (2016), and Kumar MV et al. (2018). The first experimental-guided computational prediction and mapping of hidden allosteric sites in Hsp90 combined NMR analysis, proteolytic fingerprinting and photoaffinity labeling with multiscale modeling of Hsp90 interactions and docking (Matts et al., 2011a,b). Computational predictions provided the first atomic resolution model of Hsp90 binding with the C-terminal modulator that fully satisfies the available experimental data and provide key insight for the structure-based design of allosteric Hsp90 inhibitors. In the subsequent study, a novel, computational approach for the discovery of allosteric inhibitors based on the physical characterization of signal propagation mechanisms was applied to the Hsp90 chaperone (Morra et al., 2010). By characterizing the allosteric hotspots of the inter-domain communication pathways, dynamic pharmacophore models to screen small molecules were developed. The computational predictions were combined with experimental validation showing that the selected molecules bind the allosteric sites of Hsp90, exhibit anti-proliferative activity in different tumor cell lines, and destabilize Hsp90 client proteins (Morra et al., 2010). The recent series of studies by Colombo and colleague have reported results of computer-aided design and synthesis of new allosteric ligands with micromolar to nanomolar anticancer activities, demonstrating the power of computational approaches in discovering allosteric modulators that can probe the relationships between structure dynamics and function of the Hsp90 proteins and regulatory complexes with client proteins (Sattin et al., 2015; D'Annessa et al., 2017; Masgras et al., 2017; Ferraro et al., 2019; Hu et al., 2020; Sanchez-Martin et al., 2020). Computational targeting of the Hsp90 client proteins based on the prediction of locally unstable substructures in proteins was used to develop potent probes and peptides blocking Hsp90-client interactions (Colombo et al., 2020). Recent efforts have also produced small molecules that can inhibit the inter-chaperone protein-protein interactions for Hsp70 chaperone (Gestwicki and Shao, 2019). These chemical probes have shown a considerable promise in interrogating chaperone networks in a range of models. Design, synthesis, and biological evaluation of small molecules that regulate the interaction between two Hsp70 and HOP chaperones reported the first class of compounds that specifically modulate these protein-protein interactions and inhibit protein folding events (Zaiter et al., 2019). An integrated computational and experimental approach probed allosteric mechanisms of Hsp70 binding, showing that symbiotic employment of different research tools in dissecting allosteric events in signaling proteins can be instrumental to discover selective allosteric modulators of protein functions (Rinaldi et al., 2018).

## New Developments in Modeling of Allosteric Regulation: Information-Theoretical Analysis of Dynamic Flows and Entropy Transfer in Proteins

**Figure 2:**
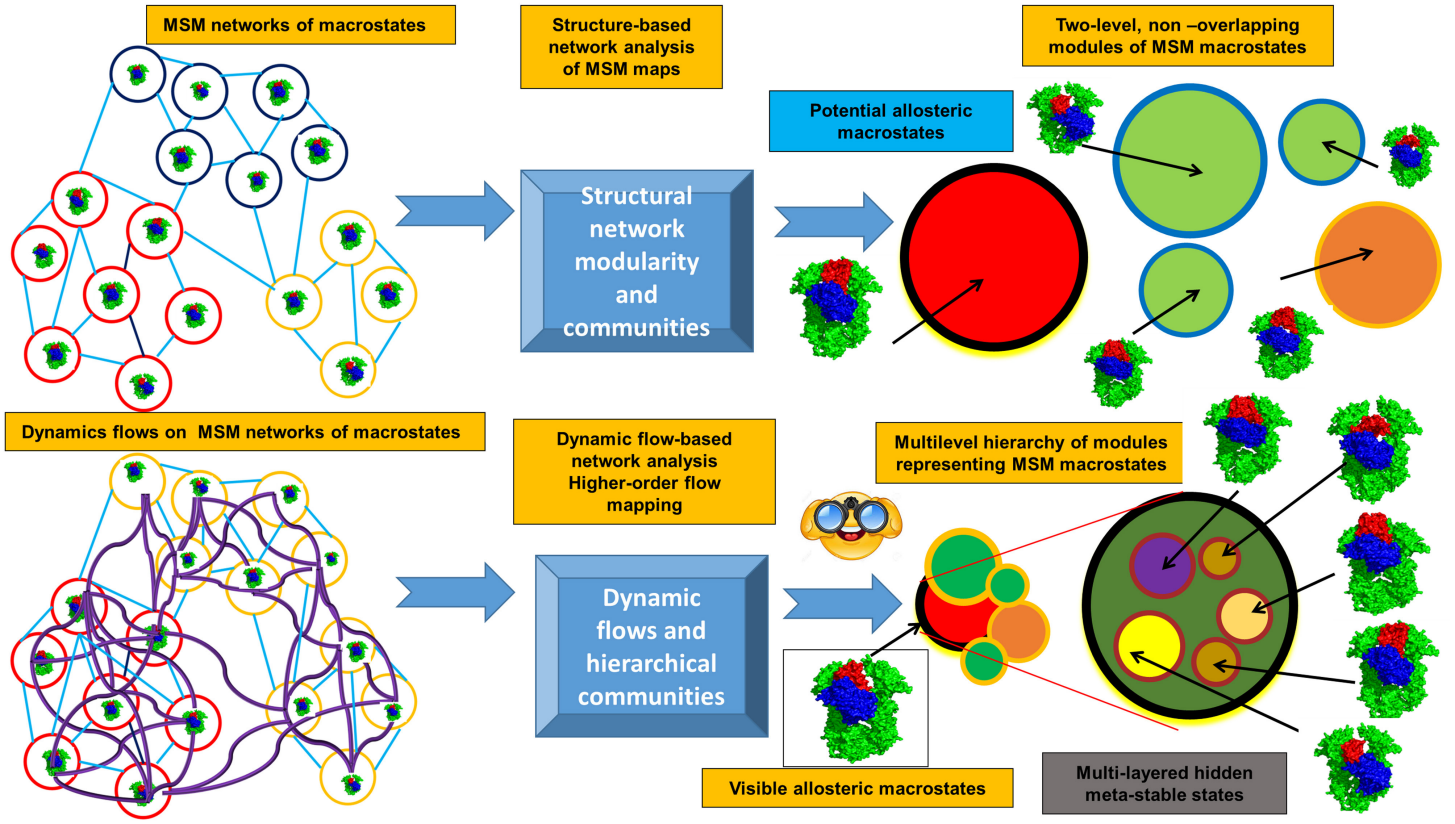
Overview of the information-theoretic framework for modeling of allosteric states and communications. The upper panel presents structure-based community detection. The lower panel illustrates modeling of the dynamical flows on the MSM maps of states and hierarchical dynamics-based detection of allosteric states and persistent communities.

The emerging computational approaches that are now employed for studies of allosteric states and mechanisms include experiment-informed network approaches, Markovian modeling and also the information-theoretical methods that model dynamic flows and entropy transfer in complex systems. By describing protein dynamics as a dynamically evolving network of interconnected modules, the topological regularities of the network structure can be identified, while filtering out the relatively unimportant details. A modular description of a network can be viewed as a compression of that network topology, and the problem of community identification can be viewed as a problem of finding an efficient compression of the network structure and topology. Using this premise, the challenge of identifying the community structure of complex networks describing dynamic energy landscapes of allosteric proteins can be reformulated as an information-theoretic approach. Flow-based model methods operate through a stochastic walk on the dynamics of the network rather than on its topological structure, where communities consist of dynamically interconverting conformations among which the dynamic flow can persist for a long time and define functionally significant states (Rosvall and Bergstrom, 2007, 2008, 2010, 2011; Lancichinetti and Fortunato, 2009; Schaub et al., 2012; Rosvall et al., 2014; Kawamoto and Rosvall, 2015). This information-theoretical analysis can quantify the structure and dynamics of the proteins from a unified perspective in which short term dynamics is integrated into a long term behavior of the system through a modular description of dynamic flows occurring on a given network (Figure 2). In this approach, a random walk is used as a proxy for the dynamic flow on the network. The map equation method implemented by the Infomap algorithm (Edler et al., 2017) can find the optimal community partition of the dynamic conformational ensembles on different time scales (derived from MD simulations or MSM maps of macrostates) and identify dynamically persistent (as opposed to topology-derived) communities of functional macrostates. This dynamic flows method compresses the flows by aggregating nodes (states) with rapid stochastic movements, revealing network regularities as distinct dynamic modules in which flows are contained on a given time scale. The map equation has been also extended to the higher-order Markov dynamics (Lancichinetti and Fortunato, 2009; Lambiotte et al., 2011, 2019; Schaub et al., 2012; Rosvall et al., 2014; Delvenne et al., 2015; Salnikov et al., 2016). NMR chemical shift perturbations have been combined with Markov modeling and information-theoretical analysis to reveal the dynamic flow of communication between allosteric communities and identify critical residue nodes within the communication pathways in protein kinases (Aoto et al., 2016).

This information-theoretical approach can also explore the dynamic evolution of the hierarchical multi-layered interaction networks and has a potential to uncover hidden allosteric states associated with the different dynamic time scales (Figure 2). Synchronization and causality are basic non-linear phenomenon observed in diverse complex systems, including allosterically regulated proteins. When studying allosteric mechanisms and communications in proteins, it is important not only to detect synchronized allosteric states, but also to identify causal relationships between them. The knowledge of information-theoretic measures is essential for the analysis of information flow between allosteric states and presents a challenging problem (Hlavácková-Schindler et al., 2007). The problem of finding a measure that is sensitive to the directionality of the flow of information has been explored using non-linear Granger causality of time series (Ancona et al., 2004). An asymmetric quantity termed Transfer Entropy (TE), has been proposed to estimate the directionality of the coupling and flow of information (Schreiber, 2000). The information-theoretic approaches measuring causal influences in multivariate time series (Hlavácková-Schindler et al., 2007; Ito, 2016; Darmon and Rapp, 2017) can be also applied to studies of allosteric protein states and mechanisms. The quantitative models of information flow between two correlated processes (Schreiber, 2000) were adopted to quantify time delayed correlations and entropy transfer between residue pairs as a measure of allosteric coupling in proteins (Hacisuleyman and Erman, 2017a,b). Through analysis of entropy transfer, one can determine residues that act as drivers of the fluctuations of other residues, thereby determining causality in the correlations and identifying residues that act as drivers of allosteric communication in proteins (Hacisuleyman and Erman, 2017a,b). The relative entropy concept from information theory was used as a quantitative metric to develop a method for measurement of the population shift during allosteric transitions (Zhou and Tao, 2019). The developed relative entropy-based dynamical allosteric network (REDAN) model was sucessfully applied to the second PDZ domain (PDZ2) in the human PTP1E protein, providing an accurate assessment of allosteic pathways (Zhou and Tao, 2019). A rigorous mathematical framework offered by the information-theoretical formalism of dynamic network flows combined with biophysical simulations may prove to be useful for finding modular patterns and dynamically persistent communities of macrostates. The integration of this methodology with NMR experiments can aid in the better identification of functional allosteric states by matching evolution of dynamic communities against the NMR chemical shift patterns and biophysical experiments.

## The Rise of the Machines: Allosteric Mechanisms through the Lens of Machine Learning

**Figure 3:**
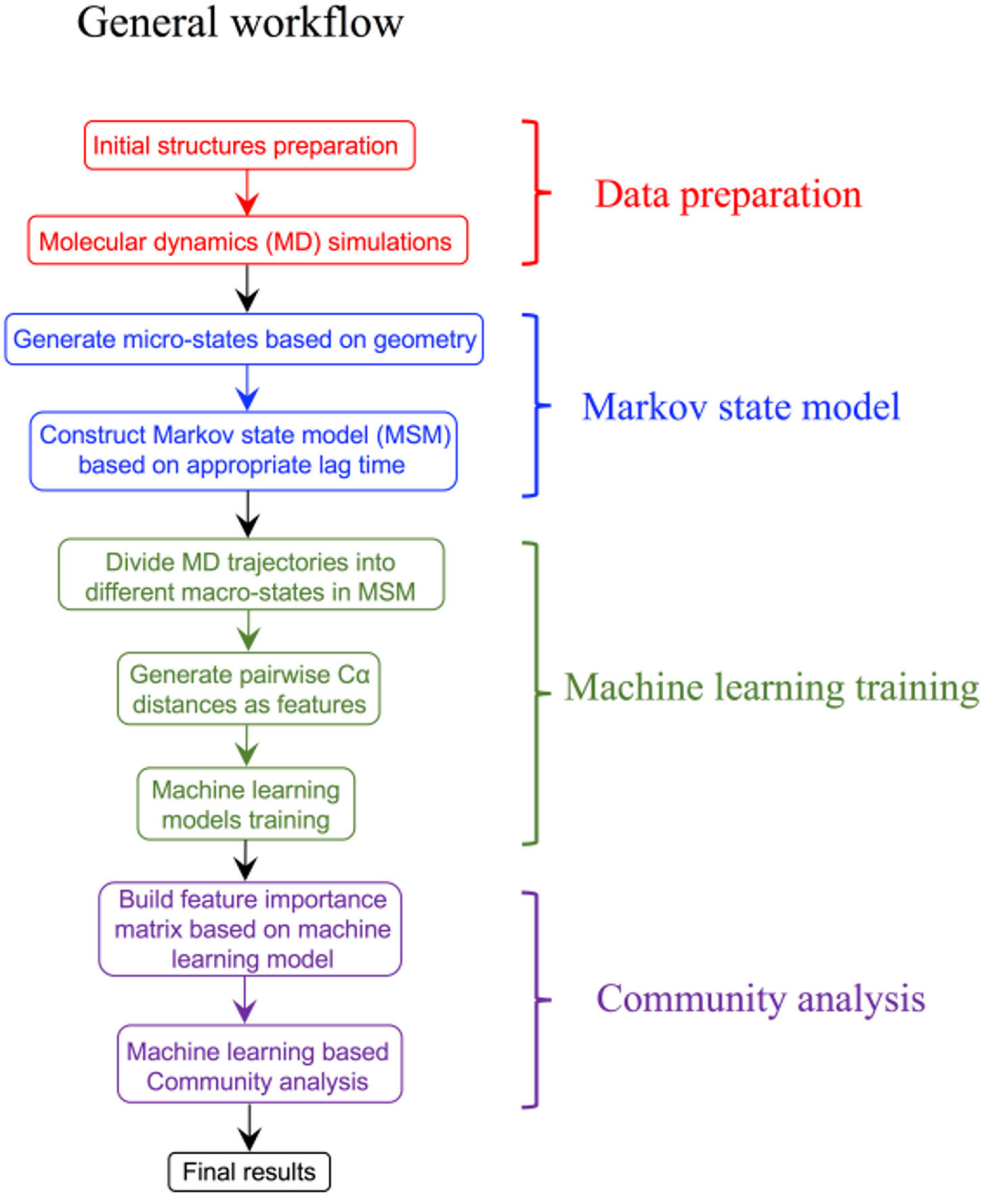
A general prototypical workflow of MSM approaches and ML modeling for detection and classification of functional allosteric states.

Over the last few years, advances in the ML field have driven the design of new computational systems that improve with experience and are able to model increasingly complex chemical and biological phenomena (Goh et al., 2017; Korotcov et al., 2017; Chen et al., 2018a; Popova et al., 2018; Dimitrov et al., 2019; Mater and Coote, 2019). ML techniques have been successfully applied to various computational chemistry challenges (Husic and Pande, 2018), pharmaceutical data analysis (Burbidge et al., 2001), protein–ligand binding affinity prediction problems (Ballester and Mitchell, 2010, Decherchi et al., 2015), dissecting molecular determinants of protein mechanisms and biochemical reactions (Li et al., 2015, Cortina and Kasson, 2018, Shcherbinin et al., 2019). Data-intensive ML modeling can be also applied for detection and classification of allosteric protein states. The integration of Markov modeling, simulations and ML approaches into robust and reproducible computational pipelines with the experimental feedback can be explored for atomistic modeling and classification of allosteric states (Figure 3). Several ML algorithms including decision tree and artificial neural networks were employed in combination with MSM approaches to develop classification models of functionally relevant allosteric conformations that exhibit very similar tertiary structures (Zhou et al., 2018a,b). Despite the lack of significant conformational change between allosteric states of the second PDZ domain (PDZ2) in the human PTP1E protein, which is a prototypical example of dynamics-driven allostery, it was demonstrated that both algorithms could build effective prediction models and provide reliable quantitative evaluation of the contributions from individual residues to the difference between the two allosteric states (Zhou et al., 2018a,b). A high prediction accuracy and sensitivity of the ML models to small structural and dynamic changes have demonstrated the utility of these approaches in probing subtle allosteric changes. Deep neural networks were used in combination with MD simulations of the PDZ3 domain of PDS-95 revealing that allosteric effects can be quantified as residue-specific properties through two-dimensional property-residue maps (Hayatshahi et al., 2019). These residue response maps could accurately describe how different protein residues are affected by allosteric perturbations exerted on the protein system. Another ML-based analysis of protein dynamics was employed to compare the binding modes of TEM-1 β-lactamase in different catalytic states (Wang et al., 2019). While conventional analysis methods including principal components analysis (PCA) could not differentiate TEM-1 in different binding modes, neural network models resulted in an excellent classification scheme that differentiated between closely related functional states (Wang et al., 2019). This study has provided a unique insight into the role and specific function of individual residues, highlighting their contributions to the delicate thermodynamic balance between allosteric states.

The remarkable rise of deep learning (DL) relying on the robust function approximations and representation properties of deep neural networks has provided us with new tools to automatically find compact low-dimensional representations (features) of high-dimensional data (LeCun et al., 2015). DL models have achieved outstanding predictive performance making dramatic breakthroughs in a wide range of applications, including automatic speech processing and image recognition (Toledano et al., 2018; Kim et al., 2019; Hey et al., 2020; Wu et al., 2020). In the words of Geoffrey Hinton who is the founder of DL technologies “Deep Learning is an algorithm which has no theoretical limitations on what it can learn; the more data you give and the more computational time you provide the better it is” (LeCun et al., 2015). Deep neural network methods were successfully applied to predict intrinsic molecular properties such as atomization energy based on simple molecular geometry and element types (Rupp et al., 2012). DL models were recently used for structure-functional prediction of cancer mutations and functional hotspots of ligand binding in cancer-associated genes (Agajanian et al., 2018). The developed models can capture ~90% of experimentally validated mutational hotspots and yield novel information about molecular signatures of driver mutations. In the recent studies, we have proposed novel DL architectures capable of predicting functional protein hotspots directly from raw nucleotide sequence information (Agajanian et al., 2019). These studies have shown that DL models can learn high importance features from raw genomic information and produce reliable recognition and classification of functionally significant cancer mutation hotspots. Moreover, these DL models can often outperform computational predictors of cancer mutations that are based on protein sequence and structure features (Agajanian et al., 2019). The success of DL tools in deciphering important functional phenotypes directly from primary sequence information is encouraging as these models can bypass the need for a large number of empirically-derived functional and structural features. However, ML methods often result in “black box” models with limited interpretability. There has been an explosion of interest in transparent and interpretable ML models to enable more efficient data mining and scientific knowledge discovery (Holzinger et al., 2014). Our investigations have also provided a roadmap how to combine DL predictions of functional sites with subsequent biophysical analysis to aid in the interpretability of ML models and facilitate their applications in biological problems (Agajanian et al., 2018, 2019).

One of the primary goals of artificial intelligence (AI) is to produce fully autonomous agents that interact with their environments to learn optimal behavior, improving over time through trial and error. An important mathematical framework for experience-driven autonomous learning through interactions with the environment is reinforcement learning (RL) (Sutton and Barto, 1981; Barto, 1994; Botvinick, 2012; Hassabis et al., 2017). While previous RL approaches lacked scalability and were limited to fairly low-dimensional problems, a marriage between deep neural networks and RL resulted in the new rapidly evolving field of deep reinforcement learning (DRL) that has achieved remarkable success in game-oriented and various scientific applications, attaining a wide popularity and celebrity-like following among researchers (Mnih et al., 2015; Silver et al., 2017; Botvinick et al., 2019; Jaderberg et al., 2019; Senior et al., 2019). DRL concepts leverage and symbiotically combine neural network modeling with reinforcement learning, in which optimization strategies are crafted based on the trade-offs and competition between rewards and punishments rather than conventional deterministic or stochastic exploration methods. After years of serving as a mere inspiration rather than a practical tool, DRL techniques have taken off overcoming scalability and data limitation issues, and exploding into one of the most intense areas of AI research. Recent years have witnessed the expansion of DRL applications into biomedical research including but not limited to biomedical informatics, drug discovery (Baskin, 2020; Grebner et al., 2020), and toxicology (Chary et al., 2020).

The rationale for employing DRL technologies in studies of allosteric regulation is to capitalize on conceptual and algorithmic similarity between Markov decisions processes (MDPs) which are at the core of RL methods and Markovian modeling of allosteric states in proteins. Several methods adopted RL-based conceptualization to develop MDP algorithms for conformational mapping of the protein landscapes and detection of functional allosteric states. REinforcement learning based Adaptive samPling (REAP) algorithm has shown a considerable promise by adopting RL principles in which an agent (or learning algorithm) takes actions in an environment (conformational protein landscape) to maximize a reward function (Shamsi et al., 2018). In this study, the action is associated with launching a pool of simulations along different collective variables (reaction coordinates), with the reward function proportional to the efficiency of a reaction coordinate to sample space and detect unknown states, and the agent selecting the directions which are most rewarding ultimately leading to the optimal adaptive strategy (Shamsi et al., 2018). Similar concepts were used to develop a goal-oriented sampling method, termed fluctuation amplification of specific traits (FAST) for rapid search of conformational space and identification of distinct functional states by balancing search near promising solutions (exploitation) and attempts to find novel solutions (exploration). Inspired by the RL ideas, this methods runs pools of simulations from starting points chosen based on the reward functions that encourages discovery of new conformations with selected physical properties (Zimmerman and Bowman, 2015; Zimmerman et al., 2018). Generative neural networks have been recently proposed as a tool for the discovery of efficient collective variables that are fundamental for adaptive exploration of the conformational landscapes and finding functional states and hidden allosteric states by guiding sampling toward poorly explored regions (Chiavazzo et al., 2017; Chen et al., 2018b; Hernandez et al., 2018; Mardt et al., 2018). MD simulations were combined with DL approach to train an autoencoder (Hinton and Salakhutdinov, 2006) in order to generate new protein conformations and mine conformational space of the bound state from an ensemble of unbound protein structures (Degiacomi, 2019). Another interesting study employed autoencoder-based detection algorithm to explore dynamic allostery induced by ligand binding based on the comparison of time fluctuations of distance matrices obtained from MD simulations in ligand-bound and unbound forms (Tsuchiya et al., 2019). In this method, the autoencoder neural network is first trained on the time fluctuations of protein motions in the apo form, and the trained autoencoder is then applied to analyze patterns of fluctuations in the holo form. Using this elegant implementation of RL approach, the authors mapped allosteric communication networks of the dynamically coupled residues and ligand pockets in the PDZ2 domain induced by binding (Tsuchiya et al., 2019). Allosteric pocket crosstalk defined as a temporal exchange of atoms between adjacent pockets in the MD trajectories can produce a fingerprint of hidden allosteric communication networks (La Sala et al., 2017). The recent RL-inspired studies of allosteric systems suggested that simulation-driven ML modeling and analysis of conformational landscapes may uncover rarely-populated functional states and shed the light on the key features of allosteric communications between visible and hidden binding pockets in proteins.

**Figure 4:**
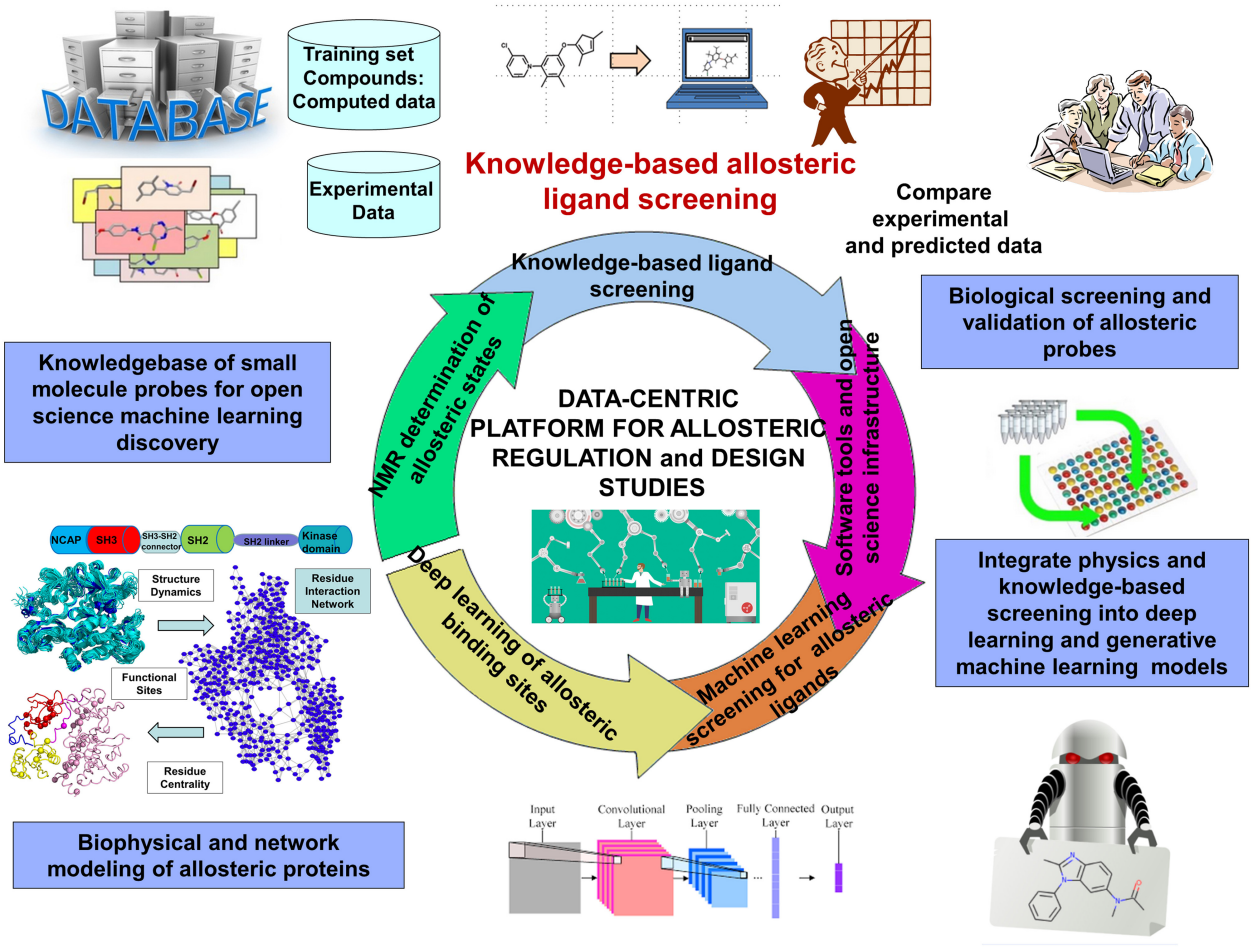
An overview of data-intensive ML platform for allosteric research and allosteric drug discovery.

DRL is a continuous trial-and-error based sampling-learning process where the agent tries to apply different combination of actions on a state to find the highest cumulative reward. Although DRL methods can tackle a wide range of learning problems with a rigorous mathematical formulation, the challenges posed by the properly crafted interplay between rich data acquisition and delayed rewards remains a significant impediment to the widespread of RL methods in many application domains, including prediction of allosteric protein states and mechanisms. The challenges of DRL approaches often lie in the art of designing robust reward function. The hybrid reward functions with a weighted combination of topological, dynamic, and network-based rewards describing different characteristics of allosteric states may represent a potentially interesting strategy to mitigate the inherent deficiencies of RL and DRL methods. For this, the rewards may be treated as neural networks trained on the set of known allosteric states. A new saga in the rapidly evolving DRL field was the development of episodic-based DRL algorithms that estimate the value of actions and states using episodic memories where the agent stores each encountered state along with the sum of rewards obtained during the n time steps (Botvinick et al., 2019). The episodic memory-based models can be extended to develop curiosity reward bonus functions for efficient exploration of the environment and detecting states in the poorly accessible regions (Han et al., 2020). In this context, DRL framework that iterates episodes of DRL and community decomposition of the dynamic network flows on the conformational landscapes may enhance the interplay between sampling and learning, thus facilitating identification of hidden allosteric states. Different from traditional DRL approaches, this strategy can consider communities of states as intermediate stages in the learning process, rather than unique states, which could potentially lead to a more robust and versatile learning procedure (Figure 4).

Deep neural network (DNN) models, most notably variational autoencoder (VAE) (Gomez-Bombarelli et al., 2018) and generative adversarial networks (GANs) (Sorin et al., 2020; Zhong et al., 2020) have proven fruitful in chemical discovery and molecular design of novel synthesizable chemical probes. Automated chemical design approaches employed VAE neural networks for a data-driven continuous representation of molecules (Gomez-Bombarelli et al., 2018). GAN models are often considered as one of the most significant advances in the field of machine learning, and their success has generated a considerable momentum with growing number of applications including molecular design of novel chemical probes and materials (Guimaraes et al., 2017; Kadurin et al., 2017a,b; Olivecrona et al., 2017; Yu et al., 2017; Gupta et al., 2018; Polykovskiy et al., 2018; Putin et al., 2018a,b) (Figure 4). By leveraging sequence data generation (SeqGAN) approach (Yu et al., 2017); Objective-Reinforced Generative Adversarial Networks (ORGAN) (Guimaraes et al., 2017) combines GANs and RL to apply the GAN framework to molecular design with domain-specific rewards and feedback. Of particular importance is MolGAN, an implicit, generative model for small molecular graphs that circumvents the need for expensive graph matching procedures and adapts GAN approach to operate directly on graph-structured data (Cao and Kipf, 2018). CycleGAN provides unpaired image-to-image translation using Cycle-Consistent Adversarial Networks (Zhu et al., 2017). MolCycleGAN, which extended the CycleGAN framework with an added loss and extra encoding network, maps from distribution to distribution on unpaired samples, so it can amplify the size of our dataset in the process by taking all of the pairing combinations rather than relying on a training dataset of predefined molecule-inhibitor pairs (Maziarka et al., 2020). The advantage of MolCycleGAN is the ability to learn transformation rules from the sets of compounds with desired and undesired values of the considered property. The methodological and algorithmic progress in GAN applications to molecular discovery has been further catalyzed by the development of several comprehensive benchmarking sets embedded into a sophisticated cheminformatics infrastructure supporting open-source implementations of molecular features and learning algorithms (Olson et al., 2017; Polykovskiy et al., 2018; Racz et al., 2019). Despite recent developments in GANs models, the applicability of these tools for molecular design continues to present a promise rather than a validated strategy, lacking systematic and comprehensive tools to target specific protein families and interrogate molecular mechanisms. There is also growing interest in generative models which can incorporate both chemical and structural information about small molecules and their interactions with protein targets.

## Synergies and Limitations of Computational Approaches for Quantitative Modeling of Allosteric Regulation

A systematic interdisciplinary effort is needed to leverage the burgeoning knowledge about allosterically regulated proteins in the development of experiment-informed data-oriented computational tools for prediction of allosteric mechanisms and allosteric drug discovery. The main advantage of experiment-informed Markovian modeling is the ability of this technique to adequately describe hierarchy of allosteric states and the molecular basis of allosteric mechanisms. Using a combination of NMR-guided simulations and MSM approach, we can determine structural and dynamic patterns of conformational ensembles and identify functional allosteric states that are hidden in the conformational ensembles. The critical challenges of these methodologies for modeling allosteric regulation phenomenon is selecting a set of experimentally-informed collective variables defined by the intrinsic dynamics to provide the optimal projection of the landscape into functional allosteric states. In this context, the newly emerging information-theoretical flow approaches and modeling of entropy transfer in proteins can represent viable complementary tools for adequate reconstruction of functional conformational landscapes in proteins. The proposed integration of biomolecular simulations and NMR experiments with machine learning into a comprehensive research platform is expected to produce a toolkit of approaches for prediction of allosteric states and mapping of allosteric mechanisms.

Network algorithms, information-theoretical approaches and DL models may be time-consuming and require a systematic exploration and engineering of features and neural net architectures with a constant and evolving feedback from NMR experiments to validate and confirm predictions. Several different ML architectures can be further explored to address potential efficiency and convergence problems including transfer learning, imitation learning, episodic control and dueling networks. To achieve synergies and robust integration of emerging technologies for predicting allosteric regulation mechanisms, a new open science infrastructure development is required which implies extensive sharing of experimental and computational data, software and knowledge across many discipline. Through integrative studies of allosteric mechanisms empowered by biophysical and data science approaches we can expand the toolkit of to dissect and interrogate allosteric mechanisms and functions in the therapeutically important protein families.

## Conclusion

The growing body of computational and experimental studies has shown that integration of data-driven biophysical and ML approaches can bring about new drug discovery paradigms, opening up unexplored venues for further scientific innovation and unique biological insights. The integration of computational and NMR approaches into a novel research platform that explores experiment-informed physical simulations, Markov state modeling, information-theoretical formalism of dynamic allosteric networks under the unified umbrella of machine learning will key to dissect molecular rules of allosteric regulation. The innovative cross-disciplinary approaches that expand the knowledge, resources and tools for studies of allosteric regulation can promote a broader usage of new technologies to understand and exploit allosteric phenomenon through the lens of chemical biology, material science, synthetic biology and bioengineering. By developing an open science infrastructure for machine learning studies of allosteric regulation and validating computational approaches using integrative studies of allosteric mechanisms, the scientific community can expand the toolkit of approaches and chemical probes for dissecting and interrogation allosteric mechanisms in many therapeutically important proteins. The development of community-accessible tools that uniquely leverage the existing experimental and simulation knowledgebase to enable interrogation of the allosteric functions can provide much needed impetus to further experimental technologies and enable steady progress.

## Author Contributions

GV, PT, and GH conceived and designed the research, analyzed the results, and wrote the manuscript. GV, SA, PT, and GH performed the research. GV wrote the final version of the manuscript and supervised the project. All authors contributed to the article and approved the submitted version.

## Conflict of Interest

The authors declare that the research was conducted in the absence of any commercial or financial relationships that could be construed as a potential conflict of interest.
